# Sarcoidosis and Obstructive Sleep Apnoea: An Intriguing Relationship

**DOI:** 10.2174/0118743064450100260420103730

**Published:** 2026-04-22

**Authors:** Ubaldo Ciccarelli, Paolo Ghelardini, Maria Chiara Pisciella, Giovanni Zezza, Angela Nervoso, Federico Giordani, Pier-Valerio Mari, Filippo Martone, Matteo Siciliano

**Affiliations:** 1 Facoltà di Medicina e Chirurgia, Università Cattolica del Sacro Cuore, 00168 Rome, Italy; 2 Unità di Medicina Interna, Ospedale San Carlo di Nancy, Rome, Italy; 3 APS-ETS, Amici contro la Sarcoidosi Italia, Bologna, Italy

**Keywords:** Obstructive sleep apnoea, Sarcoidosis, Excessive daytime sleepiness, Sleep disorders, Fatigue, CPAP therapy

## Abstract

Sarcoidosis is a systemic granulomatous disorder of unknown etiology, most commonly affecting the lungs but potentially involving multiple organs. Fatigue and Excessive Daytime Sleepiness (EDS) represent highly prevalent and disabling symptoms, often persisting despite stable systemic disease. Growing evidence indicates an increased prevalence of Obstructive Sleep Apnoea (OSA) among patients with sarcoidosis, suggesting a complex and multifactorial association.

Several mechanisms contribute to this link. Shared inflammatory pathways promote systemic inflammation and vascular dysfunction. Corticosteroid therapy, although central to sarcoidosis management, indirectly predisposes to OSA through weight gain, fat redistribution, and myopathy. Direct upper airway involvement (sarcoidosis of the upper respiratory tract, SURT) may cause mechanical obstruction, while restrictive pulmonary disease reduces lung volumes and increases pharyngeal collapsibility.

Clinical studies consistently report a higher prevalence of OSA in sarcoidosis compared with the general population, even in non-obese and treatment-naïve patients. While OSA contributes to fatigue and EDS, these symptoms are also influenced by immune dysregulation, chronic inflammation, and treatment side effects. Continuous Positive Airway Pressure (CPAP) therapy has demonstrated efficacy in reducing apnoea burden and improving both fatigue and quality of life, underscoring the importance of systematic OSA screening in sarcoidosis patients with persistent sleep-related symptoms.

Despite increasing recognition, current evidence is limited by small sample sizes, heterogeneous diagnostic methods, and confounding effects of corticosteroid therapy. Future studies should clarify pathophysiological mechanisms, evaluate the impact of OSA treatment on sarcoidosis outcomes, and investigate wake-promoting agents such as solriamfetol and pitolisant as potential adjunctive therapies in patients with residual EDS.

## 1 INTRODUCTION

Sarcoidosis is a systemic granulomatous disease of unknown etiology, characterized by the formation of non-caseating granulomas in affected organs, most commonly the lungs and intrathoracic lymph nodes. Pulmonary involvement is the most frequent manifestation, observed in over 90% of patients, and may present with symptoms such as cough, dyspnea, and chest discomfort. However, the disease can affect virtually any organ system, including the skin, eyes, liver, heart, and nervous system. Diagnosis is based on a compatible clinical and radiographic picture, histological evidence of non-caseating granulomas, and exclusion of other granulomatous diseases [1].

The annual incidence of sarcoidosis ranges from 1 to 15 cases per 100,000 people, with the highest rates reported in Scandinavian countries (11 to 15 cases). Significant regional differences exist even within countries, likely due to genetic, environmental, and diag-nostic factors. The average age at diagnosis is around 50 years, with a sex-related bimodal distribution: incidence peaks earlier in men (20 to 45 years) and later in women (50 to 65 years). Certain ethnic groups, such as Black Americans and individuals of Afro-Caribbean or North African origin, show higher incidence rates [2].

General symptoms are frequent in sarcoidosis [3]. Fatigue, detected by the Fatigue Assessment Scale (FAS), is one of the most common and disabling symptoms in sarcoidosis, reported in up to 70–90% of patients, even when the disease is clinically inactive [4]. It is typically described as a persistent, overwhelming sense of physical and mental exhaustion that is disproportionate to exertion and not alleviated by rest or sleep [5]. Fatigue and EDS in sarcoidosis frequently coexist with mood disturbances such as depression and anxiety, even though the association between psychological distress and inflammatory chronic disease is not well understood. Evidence from large population-based and cohort studies suggests that chronic physical illnesses are associated with a higher burden of psychiatric morbidity, likely mediated by shared inflammatory pathways, neuroimmune dysregulation, and psychosocial factors. This interaction may worsen symptom impact and complicate the clinical interpretation of fatigue and sleep-related complaints in sarcoidosis, independently of objective disease activity [5, 6].

The diagnosis of sarcoidosis is based on three major criteria: a consistent and adequate clinical presentation; demonstration of the presence of non-caseating granulomas in one or more tissue samples; and exclusion of other causes of granulomatous disorders [7]. The first line therapy for sarcoidosis is corticosteroid, in particular prednisone. Another approach is based on the utilization of immunosuppressants such as methotrexate, azathio-prine, and mycophenolate mofetil.

An increased incidence of Sleep-disordered Breathing (SDB), in particular Obstructive Sleep Apnoea (OSA), has been observed in patients affected by sarcoidosis [8].

OSA is a common sleep-related breathing disorder characterized by recurrent episodes of partial (hypopnea) or complete (apnoea) upper airway obstruction during sleep, leading to intermittent hypoxemia, sleep fragmentation, and marked fluctuations in intrathoracic pressure. OSA is highly prevalent in the adult population [9, 10]. According to the HypnoLaus study, 49.7% of men and 23.4% of women presented a moderate to severe OSA [11], compared to the previously reported prevalence in the general adult population, which ranged from 6% to 17% [12].

The pathophysiology of OSA is multifactorial and involves both anatomical and non-anatomical traits, commonly summarized in the PALM model: pharyngeal collapsibility, loop gain, arousal threshold, and muscle responsiveness [13].

OSA patients present two different kinds of symptoms: nocturnal and daytime symptoms.

The most frequent and characteristic nocturnal symptoms of OSAS are snoring and observed apneas.

Instead, the most frequent daytime symptom is daytime sleepiness, due to sleep fragmentation caused by recurrent awakenings that usually terminate the apneas and hypopneas. Morning headaches, apathy, depression, concentration difficulties, memory loss, and decreased libido are other characteristic daytime symptoms of patients with OSAS, all as a consequence of daytime sleepiness [14].

Since sleepiness is largely a subjective experience, its assessment relies mainly on validated self-report tools. The Epworth Sleepiness Scale (ESS) remains the work-horse instrument: eight everyday scenarios are scored 0–3 for dozing likelihood, yielding a global score (0 to 24) in which values > 10 denote pathological “trait” sleepiness and serve as both a diagnostic flag and a treatment target [15]. To assess moment-to-moment fluctuations in alertness, brief, single-item tools such as the Karolinska Sleepiness Scale (KSS) and the Stanford Sleepiness Scale (SSS) are commonly used [16, 17]. When subjective reports and clinical impression diverge (or when medico-legal or safety-critical decisions are at stake), laboratory procedures such as the Multiple Sleep Latency Test (MSLT) and Maintenance of Wakefulness Test (MWT) provide objective latencies that index physiologic sleep propensity or wake maintenance ability, respectively [18]. For OSA risk assessment, questionnaires such as the STOP-Bang and the Berlin Questionnaire provide quick, reliable screening based on symptomatology, clinical features, and anthropometric data, helping identify patients who require further diagnostic testing like polysomnography or respiratory polygraphy [19, 20]. Moreover, instruments like the Functional Outcomes of Sleep Questionnaire (FOSQ) assess the impact of sleepiness on daily functioning, aiding in comprehensive patient assessment (Table **1**) [21, 22].

The gold standard for diagnosis is Polysomnography (PSG), which records several physiological signals during sleep, such as airflow, respiratory effort, oxygen saturation, and brain activity. The Apnea-Hypopnea Index (AHI), which measures the number of apneas and hypopneas per hour of sleep, plays a key role in the diagnosis. An AHI > 5 with symptoms, and an AHI ≥ 15, even without symptoms, confirms the OSA diagnosis [23, 24].

Treatment requires a tailored approach for any condition. The first-line therapy for moderate-to-severe OSA is Continuous Positive Airway Pressure (CPAP), which keeps the upper airway open by delivering a constant stream of pressurized air *via* a nasal or oronasal mask. CPAP is highly effective in reducing apnoea events and improving quality of life, though adherence can be challenging. For mild OSA, and for patients who cannot tolerate CPAP, other approaches could include lifestyle changes and upper airway surgical approaches [25, 26].

## 2 METHODS

This narrative review was conducted through a structured literature search of PubMed and Scopus databases. The search strategy included combinations of the keywords “sarcoidosis”, “obstructive sleep apnoea”, “sleep-disordered breathing”, “fatigue”, and “excessive daytime sleepiness”. Articles published in English were considered, with priority given to original studies, systematic reviews, and meta-analyses relevant to the epidemiology, pathophysiology, and management of OSA in sarcoidosis. Reference lists of selected articles were manually screened to identify additional relevant studies.

### 2.1 Potential Mechanistic Links between OSA and Sarcoidosis

#### 2.1.1 Chronic Inflammation as a Shared Pathway

Although the link between OSA and sarcoidosis remains incompletely understood, several studies suggest that the two conditions may share overlapping inflammatory and vascular pathways. Chronic systemic inflammation has been hypothesized to represent a common feature of both diseases: intermittent hypoxia in OSA induces oxidative stress and immune activation, while sarcoidosis is characterized by granulomatous inflam-mation and dysregulated immune responses. Understan-ding these shared mechanisms may help clarify their combined impact on cardiovascular risk and fatigue, although further studies are required.

During apnoeic episodes, repeated oxygen desaturation triggers inflammatory responses involving endothelial cells, monocytes, and neutrophils, increasing oxidative stress and vascular dysfunction [27, 28]. In sarcoidosis, pre-existing endothelial dysfunction due to granulomatous vasculitis may be further influenced by the presence of OSA [27].

Patients with both conditions show a significantly elevated Augmentation Index (AIx), an indicator of arterial stiffness, which correlates positively with the Apnoea–Hypopnoea Index (AHI), suggesting a possible association between OSA severity and vascular dysfunction in sarcoidosis. Roeder *et al.* (2023) investigated arterial stiffness in sarcoidosis patients with and without Obstructive Sleep Apnoea (OSA), using the Augmentation Index (AIx) as a surrogate marker. The authors underline that patients with both sarcoidosis and OSA had significantly higher AIx values compared to those without OSA, indicating increased arterial stiffness and early vascular ageing. Notably, a positive association was observed between AHI and AIx after adjustment for confounders, including age, blood pressure, and corticosteroid use. Moreover, patients with severe OSA (AHI ≥ 30) exhibited higher AIx values, supporting the hypothesis that OSA may contribute to vascular dysfunction in sarcoidosis [29].

Shared inflammatory markers, such as IL-6, TNF-α, and CRP, are elevated in both diseases and are thought to contribute to chronic systemic inflammation and cardiovascular risk [30, 31]. Recent data also suggest that autonomic dysfunction may play a role in the symptom burden of sarcoidosis. In a study assessing Heart Rate Variability (HRV) in patients with pulmonary sarcoidosis, Mari *et al.* (2025) reported significant alterations in both time- and frequency-domain HRV parameters, indicating impaired autonomic balance. These findings highlight a potential neurocardiac mechanism contributing to fatigue and cardiovascular risk in sarcoidosis, independent of OSA [32].

Adhesion molecules such as ICAM-1 and VCAM-1 are also increased, encouraging leukocyte adhesion and endothelial dysfunction [33]. A further shared mechanism has been proposed to involve activation of NF-κB, a transcription factor responsive to oxidative stress and hypoxia, which regulates pro-inflammatory mediators and adhesion molecules [34]. Toll-like receptors (TLR-2 and TLR-4), upregulated in response to hypoxia and inflam-mation in OSA, have also been implicated in innate immune activation in sarcoidosis [35].

Another potential point of convergence is HIF-1α, which is upregulated in OSA due to recurrent desaturation and has been shown to contribute to sustained granulomatous inflammation in sarcoidosis by regulating IL-1β and IL-17 production [36-38]. Systemic and neural inflammation mediated by IL-6 and TNF-α may help explain persistent fatigue in both diseases *via* neuroinflammatory pathways [30].

#### 2.1.2 Corticosteroid-induced Obesity as a Mediator Between Sarcoidosis and Obstructive Sleep Apnoea

An additional mechanism that has been suggested in the relationship between sarcoidosis and OSA is the indirect role of corticosteroid-induced obesity. The development of Cushingoid features and weight gain is dose- and duration-dependent and may occur early during therapy, with iatrogenic Cushing’s syndrome representing a marker of increased cardiovascular risk [39, 40]. Several mechanisms contribute to corticosteroid-associated weight gain, including increased appetite and higher caloric intake related to gastrointestinal side effects [41]. Recent findings suggest that obesity is not only a frequent consequence of sarcoidosis treatment but may also influence disease activity through chronic low-grade inflammation. In obese individuals, adipose tissue is enriched in immune cells and tends to promote a Th1-skewed pro-inflammatory environment, potentially contributing to granulomatous inflammation [42-44]. Obesity is a well-established independent risk factor for OSA, as adipose tissue accumulation in the neck and peripharyngeal region may increase upper airway narrowing and collapsibility during sleep, while central obesity may impair lung volumes and neuromuscular control of breathing [45-48]. Taken together, corticosteroid-induced weight gain in sarcoidosis may indirectly increase the risk and severity of OSA, adding to cardiopulmonary burden. This potential cascade should be considered when managing long-term corticosteroid therapy.

#### 2.1.3 Upper Airway Sarcoidosis as a Direct Contributor to Obstructive Sleep Apnoea

A more direct anatomical mechanism that has been proposed is upper airway involvement in the form of Sarcoidosis of the Upper Respiratory Tract (SURT). SURT occurs in approximately 5% of sarcoidosis patients and may be associated with granulomatous infiltration of the nasal passages, larynx, pharynx, and epiglottis, leading to mechanical airway narrowing [49-51]. These changes may increase airway collapsibility during sleep, a core pathophysiological feature of OSA. Case reports have described sarcoid-related epiglottic inflammation associated with severe OSA, with improvement following immunosuppressive therapy, supporting a possible causal relationship in selected cases [52]. SURT frequently coexists with other sarcoidosis-associated risk factors for OSA, including glucocorticoid-induced weight gain and steroid-related myopathy, which may further compromise upper airway patency [40, 42]. Lal *et al.* (2015) reviewed several mechanisms linking sarcoidosis and sleep-disordered breathing, supporting the thesis that OSA in sarcoidosis likely results from a combination of anatomical and systemic factors [8].

#### 2.1.4 Volume Loss in Restrictive Lung Disease: A Mechanical Link to OSA

Another proposed mechanism involves the effect of restrictive lung disease on upper airway mechanics. In interstitial lung diseases, including pulmonary sarcoidosis, reduced lung volumes may lead to diminished caudal traction on the upper airway, thereby increasing collapsibility during sleep [53-55]. Several epidemiological studies have reported a higher prevalence of OSA among patients with ILDs, particularly Idiopathic Pulmonary Fibrosis (IPF), in which OSA has been observed in up to 60–80 % of cases [56, 57]. Bingöl *et al.* (2015) evaluated 29 sarcoidosis patients and reported a higher oxygen desaturation index in those with parenchymal lung involvement, suggesting that pulmonary disease severity may influence OSA burden [58].

### 2.2 Clinical Evidence Supporting the Association

Several clinical and observational studies have provided evidence supporting the coexistence of OSA and sarcoidosis, suggesting a complex and multifactorial association between the two conditions.

Recently, Roeder *et al.* (2022), through the OSASA study, conducted a cross-sectional comparison between patients with sarcoidosis and matched healthy controls, revealing a significantly increased prevalence of Obstructive Sleep Apnoea (OSA) among the sarcoidosis group. Specifically, 45% of sarcoidosis patients demonstrated an Apnea-Hypopnea Index (AHI) ≥ 5, indicative of OSA, compared to 31% in controls. Moderate OSA (AHI ≥ 15 events/h) and severe OSA (AHI ≥ 30 events/h) were found in 13% patients with sarcoidosis *vs.* 8% controls, and in 3% patients with sarcoidosis *vs.* 1% control, respectively. Despite this elevated prevalence, OSA alone did not fully explain the higher levels of Excessive Daytime Sleepiness (EDS) and fatigue observed in sarcoidosis patients. Indeed, sarcoidosis subjects exhibited significantly greater daytime sleepiness, as measured by the Epworth Sleepiness Scale, and increased fatigue scores on the Fatigue Assessment Scale, alongside poorer sleep-related quality of life (assessed *via* the Functional Outcomes of Sleep Questionnaire) [59]. These findings suggest that while OSA is common in sarcoidosis and likely contributes to symptom burden, additional disease-specific factors, including systemic inflammation and granulomatous involvement, may also play critical roles in driving fatigue and sleep disturbances in this population.

In a separate investigation, Ataoğlu *et al.* (2022) conducted a study involving 60 patients with sarcoidosis who underwent clinical and polysomnographic evaluations to assess the presence of OSA. The prevalence of OSA was notably high, with 70% of patients diagnosed with polysomnography. Importantly, the frequency of OSA correlated with sarcoidosis stage, increasing from 39% in stage 1 patients to 100% in those with stage 3 disease, suggesting a link between the extent of pulmonary involvement and OSA risk. Patients with OSA were also significantly older and had higher Body Mass Index (BMI) compared to those without OSA. Moreover, corticosteroid treatment and advanced lung involvement were identified as independent risk factors for OSA in this cohort [60].

Therapeutic effects have been addressed in a prospective study by Mari *et al.* (2020), who evaluated the impact of Continuous Positive Airway Pressure (CPAP) treatment on fatigue in sarcoidosis patients diagnosed with Obstructive Sleep Apnoea (OSA). Their cohort included patients with confirmed sarcoidosis and moderate to severe OSA, who were treated with CPAP over a 3-month period. The study found that fatigue, assessed by validated scales such as the FAS, significantly decreased following CPAP therapy. The mean FAS score decreased by 6.3 points (ΔFAS = –6.3), corresponding to approximately a 25 % reduction *versus* baseline fatigue. Additionally, daytime sleepiness significantly improved, with ESS scores decreasing from 6.8 to 4.0 (ΔESS = –2.8), representing a 41% reduction. Importantly, the reduction in fatigue correlated strongly with a decrease in the AHI, which dropped from an average of 28 events/hour pre-treatment to under 10 events/hour post-treatment. These findings highlight that effective management of sleep-disordered breathing through CPAP can significantly improve fatigue symptoms that are otherwise resistant to conventional sarcoidosis therapies. The study emphasizes the clinical importance of screening sarcoidosis patients for OSA, particularly those with persistent fatigue and excessive daytime sleepiness despite well-controlled systemic disease. Early identification and treatment of OSA may represent a valuable tool in the multidisciplinary management of sarcoidosis, improving patients’ quality of life and functional status [61].

Further evidence comes from Doğan *et al.* (2020), who studied 46 patients with stage I–II sarcoidosis who had never received corticosteroid or immunosuppressive treatment. They found that 28 patients (60.9%) had obstructive sleep apnoea (OSA), with 67.8% of cases classified as mild, 21.4% moderate, and 10.7% severe. REM-related OSA was present in 14.2% of cases. Surprisingly, only one patient had excessive daytime sleepiness (ESS ≥10), and 36.9% had Poor Sleep Quality (PSQI >5). Age was the only significant predictor of OSA (*p* = 0.048); BMI, lung function, and sarcoidosis stage were not associated. Notably, no patients showed signs of RLS or PLMS. These findings stress the importance of OSA screening in early-stage sarcoidosis, even in the absence of typical symptoms or risk factors [62].

Collectively, these studies provide strong clinical evidence of the high prevalence of OSA in sarcoidosis patients, independent of corticosteroid therapy or obesity alone. The coexistence of these conditions complicates symptom interpretation, particularly concerning fatigue and daytime sleepiness, and highlights the importance of routine screening for sleep disorders in this patient group to optimize management and improve outcomes.

### 2.3 Diagnostic and Therapeutic Implications

#### 2.3.1 Screening for OSA in Sarcoidosis Patients

Given the high prevalence of OSA among sarcoidosis patients and the considerable symptom overlap between the two conditions (particularly fatigue and EDS), systematic screening for OSA should be considered as part of routine clinical evaluation. Validated questionnaires, such as the ESS and the STOP-Bang questionnaire, are useful first-line tools for identifying patients at risk [63]. Moreover, portable sleep monitoring devices (polygraphy) offer a practical and accessible alternative, especially for patients with chronic respiratory diseases like sarcoidosis, facilitating diagnosis and earlier identification of sleep-disordered breathing [59].

#### 2.3.2 Fatigue and EDS in Sarcoidosis: A Clinical Gray Zone

Fatigue is one of the most prevalent and disabling symptoms reported by patients with sarcoidosis, affecting up to 50–80% of individuals regardless of disease stage or organ involvement [5]. Unlike fatigue experienced during acute illness or exertion, sarcoidosis-related fatigue is often chronic, disproportionate to physical activity, and poorly responsive to standard anti-inflammatory therapy. Its etiology is multifactorial, involving immune dysregu-lation, chronic inflammation, sleep disturbances, corticos-teroid side effects, and psychological components such as depression or anxiety [6]. Importantly, fatigue in sarcoidosis does not always correlate with objective markers of disease activity, which makes it challenging to manage and recognize in clinical practice [64]. Tools such as the FAS have been validated to quantify fatigue in sarcoidosis and are commonly used in both clinical and research settings [4]. Despite its prevalence and impact, fatigue is frequently under-recognized and undertreated, often dismissed as a non-specific symptom. However, its presence significantly impairs quality of life and may persist even in patients with clinically inactive disease, highlighting the need for targeted diagnostic and therapeutic strategies.

Frequently, fatigue overlaps with EDS, a symptom more specifically associated with sleep disorders such as OSA, which is commonly observed in sarcoidosis patients [59]. This symptomatic overlap poses a significant diagnostic challenge and often results in under-recognition of coexisting sleep-disordered breathing.

Therefore, validated tools for assessing EDS and screening for OSA are essential in clinical practice. ESS is widely used to quantify daytime sleepiness by measuring the likelihood of dozing in various daily situations; scores above 10 suggest pathological sleepiness [61, 65].

#### 2.3.3 Role of CPAP Therapy in Sarcoidosis Patients with OSA

CPAP represents the first-line treatment for moderate-to-severe obstructive sleep apnoea [24, 25]. A CPAP system consists of a pressure generator connected *via* tubing to a nasal or oronasal mask, often combined with a humidifier to improve patient comfort. By delivering a continuous stream of positive air pressure during sleep, CPAP prevents upper airway collapse, reduces the apnea-hypopnea index, and improves nocturnal oxygenation and sleep continuity [66].

From a therapeutic perspective, CPAP has shown relevant clinical benefits in patients with sarcoidosis and coexisting OSA [67]. In addition to improving classic OSA-related symptoms, CPAP therapy has been associated with a significant reduction in excessive daytime sleepiness and fatigue. In a prospective study, Mari *et al.* demonstrated that CPAP treatment led to a clinically meaningful improvement in sarcoidosis-related fatigue, a symptom that markedly impairs quality of life in this patient population [60, 61]. These findings support the role of CPAP not only in the management of sleep-disordered breathing but also as a valuable intervention to alleviate sarcoidosis-associated fatigue.

**Fig. (1):**
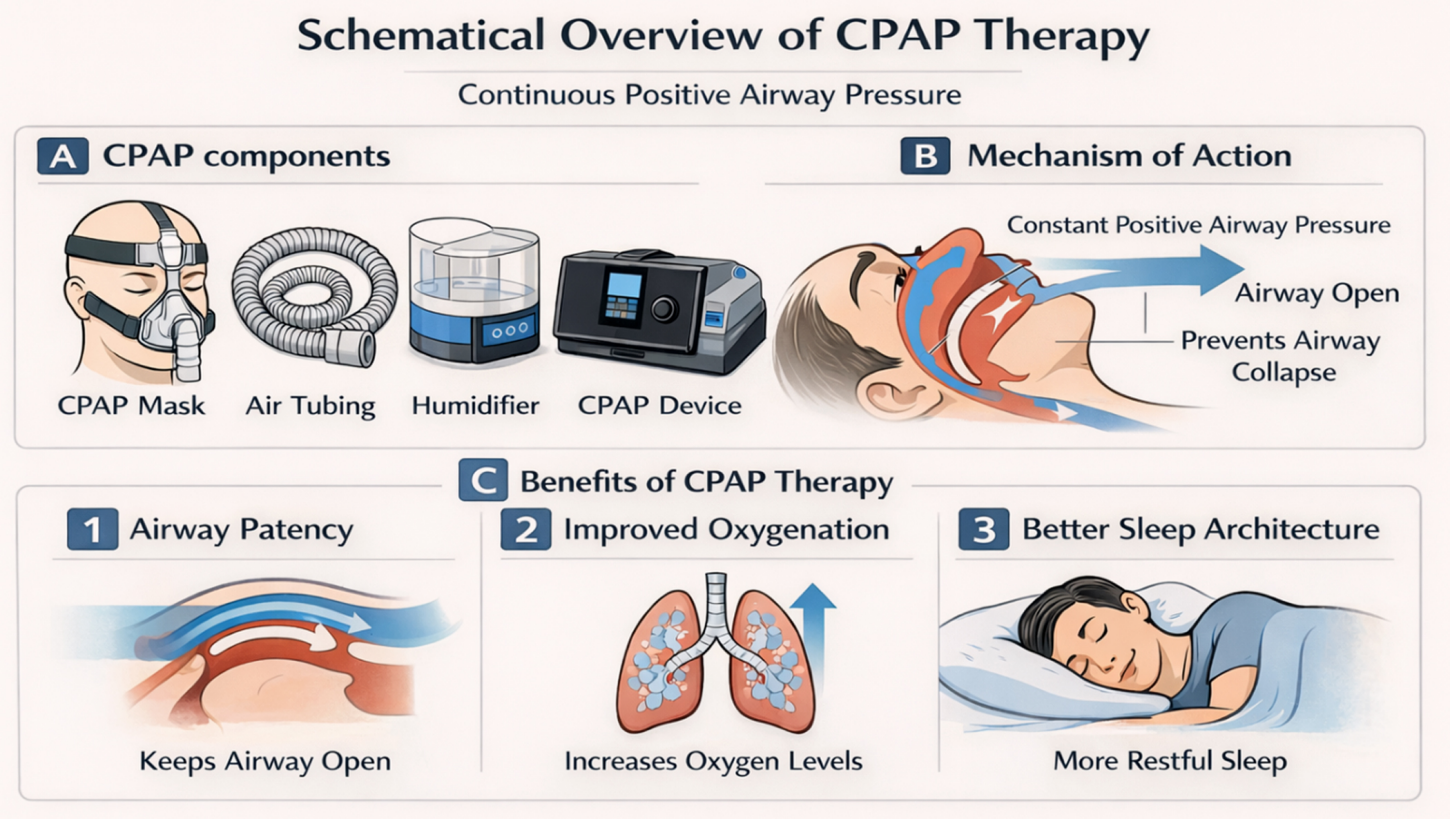
Schematic overview of continuous positive airway pressure (CPAP) therapy. ** (A) ** Main components of a standard CPAP system, including the mask interface, air tubing, humidifier, and pressure-generating CPAP device. ** (B) ** Mechanism of action of CPAP, delivering continuous positive airway pressure to the upper airway, thereby splinting the pharynx and preventing airway collapse during sleep. ** (C) ** Physiological and sleep-related effects of CPAP therapy, including maintenance of upper airway patency, improvement of nocturnal oxygenation, and restoration of normal sleep architecture.

Common side effects of CPAP therapy include nasal dryness, mask discomfort, air leaks, and claustrophobia, which may negatively affect adherence. In most cases, these issues can be effectively managed through appropriate patient education, mask fitting, and device adjustment. While fixed CPAP remains the standard first-line approach, alternative positive airway pressure modalities, including auto-adjusting CPAP and bilevel positive airway pressure, may be considered in selected patients according to clinical characteristics and tolerance (Fig. **1** and Table **2**) [68, 69].

#### 2.3.4 Impact of Steroids/Immunosuppressive Therapies on OSA

Immunosuppressive therapies, including corticos-teroids, methotrexate, TNF-α inhibitors, and mycopheno-late mofetil, play a central role in the management of sarcoidosis [64, 70]. However, corticosteroids may contribute to weight gain, muscle weakness, and conse-quently increased upper airway collapsibility, potentially exacerbating or predisposing to OSA [40]. While steroid-sparing agents such as methotrexate and mycophenolate mofetil are effective in controlling inflammation with fewer metabolic side effects, data on their direct impact on OSA are limited. Some studies have reported the efficacy of mycophenolate mofetil in sarcoidosis, especially in patients intolerant to other immun-osuppressants, with a favorable safety profile [71, 72]. Similarly, TNF-α inhibitors may reduce granulo-matous inflammation affecting the upper airways, but their effect on sleep-disordered breathing requires further investigation.

#### 2.3.5 Wake Stimulants in Sarcoidosis?

In recent years, there has been growing interest in the use of wake-promoting agents to manage persistent Excessive Daytime Sleepiness (EDS), particularly when these symptoms remain despite optimal management of the underlying disease. While earlier studies explored the off-label use of modafinil and armodafinil with some promising results in reducing sarcoidosis-related fatigue and improving quality of life, the need for newer and more effective agents has led to attention toward wake stimulants [5, 73, 74].

Solriamfetol is a dual Norepinephrine-Dopamine Reuptake Inhibitor (NDRI) approved for the treatment of EDS in both OSA and narcolepsy. Its efficacy was demonstrated in large randomized controlled trials such as the TONES 3 study, which reported that solriamfetol significantly improved wakefulness, as measured by the MWT, and reduced sleepiness scores on the ESS in patients with OSA [75]. At doses of 75 mg and 150 mg, patients showed clinically meaningful improvements in both objective and subjective measures of wakefulness, with effects seen as early as one week into treatment and sustained throughout the study duration.

Although no trials have directly evaluated solriamfetol in sarcoidosis patients, the overlapping symptom burden (particularly EDS secondary to untreated or partially treated OSA) makes it a potentially valuable therapeutic option.

Following the encouraging results with solriamfetol, attention has also turned to pitolisant, a novel wake-promoting agent with a distinct pharmacological mechanism and a potentially favourable safety profile in complex, multisystem disorders such as sarcoidosis.

Pitolisant is a selective histamine H_3_ receptor antagonist/inverse agonist, which promotes wakefulness by enhancing histaminergic neurotransmission and, indirectly, dopaminergic and cholinergic activity in the central nervous system. Unlike solriamfetol or modafinil, pitolisant does not act by inhibiting monoamine reuptake and is not classified as a controlled substance, reflecting its low abuse potential and minimal sympathomimetic activity (a relevant consideration in patients with cardiovascular or metabolic comorbidities often seen in sarcoidosis).

Its clinical efficacy in OSA-related EDS has been demonstrated in three randomized controlled trials (HAROSA I, II, and III), which showed that pitolisant significantly reduced subjective sleepiness (measured by the Epworth Sleepiness Scale) and improved sustained attention compared to placebo in patients with moderate-to-severe OSA, including those adherent to CPAP therapy but with persistent symptoms [76-78]. These results were confirmed in subsequent meta-analyses, which found consistent improvements in both EDS and fatigue outcomes without significant adverse effects, particularly regarding cardiovascular parameters [79].

Although wake-promoting agents such as solriamfetol and pitolisant have demonstrated efficacy in reducing excessive daytime sleepiness in patients with obstructive sleep apnoea, their role in sarcoidosis remains largely unexplored. To date, no clinical trials have evaluated these agents in sarcoidosis-specific populations, and their potential benefits cannot be extrapolated beyond OSA-related sleepiness.

In this context, wake-promoting agents should not be considered established therapeutic options for sarcoidosis-associated fatigue or sleepiness, but rather theoretical adjuncts that warrant investigation in carefully designed prospective studies. Their use, if any, should be restricted to selected patients with objectively documented OSA, persistent excessive daytime sleepiness despite optimal CPAP adherence, and after exclusion of other sarcoidosis-related contributors.

Future research is needed to clarify whether these pharmacological strategies offer clinically meaningful benefits in sarcoidosis or whether fatigue and sleepiness in this population predominantly reflect disease-specific inflammatory and neuroimmune mechanisms (Table **3**).

### 2.4 Challenges and Controversies

#### 2.4.1 Limitations in Current Evidence

Although the association between sarcoidosis and OSA has been increasingly investigated, the current literature presents several important limitations. First, most available studies are cross-sectional or retrospective, often with small sample sizes and a lack of adequate control groups, which limits the generalizability and strength of the conclusions. Additionally, there is considerable heterogeneity in the diagnostic tools used for OSA (ranging from self-reported questionnaires to home-based polygraphy), rather than standardized overnight polysomnography, the gold standard for diagnosis. This methodological variability complicates the comparison between studies and may lead to underdiagnosis or misclassification of OSA severity.

Another major challenge is the confounding effect of corticosteroid therapy. Many patients included in prior studies were receiving systemic steroids, which are known to influence weight, fat distribution, muscle function, and upper airway dynamics factors directly impacting OSA risk. As a result, it remains unclear to what extent sarcoidosis itself, independent of its treatment, contributes to the development of sleep-disordered breathing. Moreover, sarcoidosis-related fatigue and EDS frequently overlap but may have distinct etiologies, making differential diagnosis difficult without objective sleep studies.

Finally, there is a lack of prospective studies assessing the impact of OSA treatment (particularly CPAP) on sarcoidosis-related outcomes such as fatigue, quality of life, pulmonary function, and systemic inflammation. Similarly, data on the role of immunosuppressive therapies or wake-promoting agents in patients with coexisting sarcoidosis and OSA are sparse and anecdotal. These gaps highlight the need for high-quality, longitudinal studies to clarify pathophysiological mechanisms, refine diagnostic strategies, and evaluate therapeutic interventions in this unique patient population.

## CONCLUSION

Despite increasing awareness of sleep-disordered breathing in systemic diseases, obstructive sleep apnoea remains frequently underdiagnosed in sarcoidosis. This under-recognition is partly due to the attribution of overlapping symptoms, such as fatigue and excessive daytime sleepiness, to the underlying inflammatory disease or its treatment, without sufficient evaluation for comorbid OSA. As highlighted throughout this review, multiple studies have demonstrated a high prevalence of OSA in sarcoidosis patients, even in the absence of classic risk factors such as obesity or corticosteroid exposure. Nevertheless, routine sleep assessments are seldom integrated into the diagnostic work-up of these individuals. This gap may delay appropriate treatment and contribute to persistent symptoms, increased cardio-vascular risk, and reduced quality of life.

From a clinical perspective, a multidisciplinary approach that includes systematic sleep evaluation (parti-cularly in patients with refractory fatigue or unexplained excessive daytime sleepiness) should be considered an essential component of comprehensive sarcoidosis care. In this context, sleep-targeted interventions, primarily continuous positive airway pressure therapy in patients with documented OSA, represent the cornerstone of man-agement. Emerging pharmacological strategies, including wake-promoting agents, should not be regarded as established therapeutic options in sarcoidosis, but rather as areas of future investigation.

In addition, future research may benefit from emerging digital health and artificial intelligence-based approaches, which have shown promise in enhancing diagnostic accuracy and clinical decision support in complex medical conditions [80, 81]. Such tools could potentially assist in disentangling overlapping symptoms such as fatigue and excessive daytime sleepiness and support more personalized diagnostic and therapeutic strategies in patients with sarcoidosis and coexisting OSA. Further studies are required to clarify the role of these strategies in sarcoidosis-specific populations and to determine whether persistent fatigue and sleepiness predominantly reflect disease-related inflammatory and neuroimmune mechanisms rather than residual sleep-disordered breathing alone.

## Figures and Tables

**Table 1:** Summary of commonly used subjective and objective tests for the evaluation of sleepiness.

Questionnaire/Test	Type	Format / Items	Score Range	Indicative Threshold	Main Use
Epworth Sleepiness Scale (ESS)	Subjective (trait)	8 scenarios rated 0–3	0–24	>10 indicates pathological sleepiness	Initial screening; therapy monitoring
Karolinska Sleepiness Scale (KSS)	Subjective (state)	Single item, scale 1–9	1–9	≥7 indicates high momentary sleepiness	Tracking diurnal variations
Stanford Sleepiness Scale (SSS)	Subjective (state)	Single item, scale 1–7	1–7	≥4 clinically significant sleepiness	Rapid assessment of current state
Multiple Sleep Latency Test (MSLT)	Objective	4–5 standardized naps post-PSG	Mean latency in minutes	<8 min indicates pathological sleep propensity	Objective confirmation when needed
Maintenance of Wakefulness Test (MWT)	Objective	4 × 40 min passive wake attempts	Mean latency in minutes	<20–30 min indicates impaired alertness	Evaluate wake-maintenance ability for safety

**Table 2:** Positive airway pressure modalities and their role in the management of obstructive sleep apnoea.

Device	Pressure Delivery	Main Indications	Utility in OSA
CPAP	Fixed continuous positive airway pressure	First-line treatment for moderate-to-severe OSA	Reduces apnoea–hypopnoea index, improves nocturnal oxygenation, and decreases excessive daytime sleepiness
Auto-CPAP (APAP)	Automatically adjusts pressure based on airflow and airway resistance	Patients with variable pressure requirements or intolerance to fixed CPAP	Improves comfort and adherence while maintaining effective control of OSA and EDS
BiPAP	Two pressure levels (higher inspiratory and lower expiratory pressure)	OSA associated with hypoventilation, high pressure requirements, or CPAP intolerance	Useful in selected patients; improves ventilation, sleep quality, and daytime sleepiness

**Table 3:** Pharmacological agents investigated or potentially applicable for the management of sarcoidosis-associated fatigue and excessive daytime sleepiness.

Drug	Mechanism of Action	Evidence in Sarcoidosis	Reported Benefits	Limitations/Notes
Modafinil	Dopamine reuptake inhibitor	Small case series and open-label studies	Improved alertness, reduced fatigue	Off-label use; potential for side effects (headache, anxiety)
Armodafinil	R-enantiomer of modafinil	Limited evidence; extrapolated from modafinil studies	Longer half-life than modafinil	Less studied in sarcoidosis specifically
Solriamfetol	Dopamine/norepinephrine reuptake inhibitor (DNRI)	No current studies in sarcoidosis; approved for OSA-related EDS (Sunosi^®^ by Jazz Pharma)	FDA-approved for OSA-related EDS; rapid onset	Not yet tested in sarcoidosis trials
Pitolisant	Histamine H3 receptor antagonist/inverse agonist	No direct studies; approved for narcolepsy and OSA-related EDS	Potential benefit in central hypersomnolence syndromes	No published evidence in sarcoidosis

## LIST OF ABBREVIATIONS

EDS: Excessive Daytime Sleepiness
OSA: Obstructive Sleep Apnoea
CPAP: Continuous Positive Airway Pressure
MSLT: Multiple Sleep Latency Test
